# Beneficial Effects of Capybara Oil Supplementation on Steatosis and Liver Apoptosis in Obese Mice

**DOI:** 10.1155/2024/7204607

**Published:** 2024-05-27

**Authors:** Luciana Lontro Alves, Priscila Gomes Pereira, Bianca Torres Ciambarella, Miguel Porto Campos, Kíssila Rabelo, Ana Lúcia Rosa Nascimento, Raíssa Leal de Carvalho dos Santos Cunha, Cherley Borba Vieira Andrade, Alan Cesar Nunes Moraes, Andressa Bernardi, Fernanda Verdini Guimarães, Jemima Fuentes Ribeiro da Silva, Jorge José de Carvalho

**Affiliations:** ^1^Ultrastructure and Tissue Biology Laboratory, Institute of Biology, Rio de Janeiro State University, Rio de Janeiro, Brazil; ^2^Interdisciplinary Laboratory of Medical Research, Oswaldo Cruz Foundation, Rio de Janeiro, Brazil; ^3^Electron Microscopy Laboratory of Biology Institute, Federal Fluminense University, Rio de Janeiro, Brazil; ^4^Inflammation Laboratory, Oswaldo Cruz Foundation, Rio de Janeiro, Brazil

## Abstract

Obesity is a complex chronic disease characterized by excess body fat (adipose) that is harmful to health and has been a major global health problem. It may be associated with several diseases, such as nonalcoholic fatty liver disease (NAFLD). Polyunsaturated fatty acids (PUFA) are lipid mediators that have anti-inflammatory characteristics and can be found in animals and plants, with capybara oil (CO) being a promising source. So, we intend to evaluate the hepatic pathophysiological alterations in C57Bl/6 mice with NAFLD, caused by obesity, and the possible beneficial effects of OC in the treatment of this disease. Eighteen 3-month-old male C57Bl/6 mice received a control or high-fat diet for 18 weeks. From the 15th to the 18th week, the animals received treatment—through orogastric gavage—with placebo or free capybara oil (5 g/kg). Parameters inherent to body mass, glucose tolerance, evaluation of liver enzymes, percentage of hepatic steatosis, oxidative stress, the process of cell death with the apoptotic biomarkers (Bax, Bcl2, and Cytochrome C), and the ultrastructure of hepatocytes were analyzed. Even though the treatment with CO was not able to disassemble the effects on the physiological parameters, it proved to be beneficial in reversing the morphological and ultrastructural damage present in the hepatocytes. Thus, demonstrating that CO has beneficial effects in reducing steatosis and the apoptotic pathway, it is a promising treatment for NAFLD.

## 1. Introduction

Obesity is a chronic disease characterized by excess body fat that is harmful to health, increasing the risk of long-term medical complications and shortening the patient's life [1]. One of the main causes of obesity is due to the absorption of energy significantly greater than what the body can expend [2], not necessarily being linked to higher food intake but related to the food source and the nutritional quality of what is consumed in the diet [3]. In the last three decades, the prevalence of obesity has increased worldwide, reaching epidemic proportions and being declared by the World Health Organization (WHO) as the biggest health problem in adults worldwide [4]. According to the 2023 World Obesity Atlas from the World Obesity Federation (WOF), more than 2.6 billion people worldwide were obese or overweight in 2020, accounting for approximately 38% of the world's population. The WOF estimates that by 2035, this number will increase to 4 billion, representing more than 50% of the population [5]. Its prevalence is worrying since it is associated with the development of numerous secondary diseases, such as nonalcoholic fatty liver disease (NAFLD) [6].

Overweight and obese people have increased levels of hepatic lipids (which do not have the function of storing fat) and develop hepatic steatosis, a condition linked to NAFLD [6, 7]. NAFLD has become one of the leading causes of chronic liver disease [8], and it is an umbrella term that is typically used to describe liver conditions ranging from steatosis, such as nonalcoholic fatty liver, to fatty liver, followed by fibrosis or cirrhosis [9]. The prevalence of NAFLD has increased significantly in recent years, being approximately 25.3% in 1990–2006 and 38.0% in 2016–2019. It is believed that it affects 25% of the global population and that 20% of patients with this pathology may progress to nonalcoholic steatohepatitis (NASH) [10, 11]. Studies indicate that changes in lipid metabolism are directly involved in the process of development and progression of NAFLD, which may affect reactive oxygen species (ROS), mitochondria, endoplasmic reticulum (ER), and nicotinamide adenine phosphate (NADPH) oxidase [12]. The increased generation of ROS can result in several changes in the body, including insulin resistance (IR) and the activation of key enzymes of lipid metabolism [13].

The most effective way to treat both obesity and liver changes involves changes in the lifestyle and diet of patients with these comorbidities. However, only a small percentage of these patients manage to implement these measures to become effective [6]. Thus, the search for new therapeutic options, including pharmacological options, has been growing more and more with the use of polyunsaturated FAs (PUFA). It has already been determined that lipoxins, lipid mediators present in PUFA, can improve histopathological changes in rat livers, reducing areas of hepatic necrosis with infiltration of inflammatory cells and generating a decrease in the accumulation of hepatic lipids [14, 15]. EPA and DHA also showed several beneficial effects, such as decreasing the risk of cardiovascular syndromes and preventing the progression of IR and obesity [16].

Currently, the best-known and most used PUFA supplementation is fish oil [17]. However, seasonal variation, marine pollution, and increased commercialization of omega-3 have created a high demand for this oil, making its use somewhat unsustainable [18]. Both this and other more common extraction sources (plants and animals) have a complex process to obtain and generate a small amount of the final product [19]. Studies related to medicinal aspects and the use of natural resources have become increasingly common. Initially, studies were focused on plants with medicinal aspects; however, some researchers have already been using the fauna as study targets [20]. Therefore, alternative sources for obtaining these fatty acids (FAs) are essential, such as capybara oil, which would be extremely interesting in this scenario.

There are still few studies in the literature on capybara oil. This oil is extracted from the subcutaneous fat of the capybara. It contains 17.9% of *α*-linolenic FAs and 19.6% of linoleic FAs [21], which correspond to omegas 3 and 6, respectively, in addition to 35.6 to 39.8% of oleic PUFA and 20.7 to 24.3% of palmitic PUFA [22]. It is one of the components present in Capivarol, a fortifying tonic for children, and it is also related to activities in helping respiratory diseases such as bronchitis and asthma, as well as rheumatism [23]. Previous studies by our group demonstrate that it can accelerate the healing process of skin wounds in rats [24], improve steatosis, inflammation, and hepatic mitochondrial activity [25], and improve the pathophysiology and renal inflammation of obese mice [26].

Based on this information, we intend to evaluate the hepatic pathophysiological alterations in C57Bl/6 mice with NAFLD, caused by obesity, and the possible beneficial effects of OC in the treatment of this disease.

## 2. Materials and Methods

### 2.1. Obtaining Capybara Oil

Capybara oil was obtained from the “Santa Luzia Farm” for wild and exotic animals in Goias State, Brazil. The animals were raised in captivity, authorized by “The Brazilian Institute of the Environment and Renewable Natural Resources” (IBAMA). The oil extraction was performed as previously described in Marinho et al. [24] through the hydrothermal processing of the fat in a water bath for liquid oil for oral treatment of mice.

### 2.2. Experimental Groups and Diets

All experimental protocols performed were approved by the Committee for Ethics in Animal Experimentation of the State University of Rio de Janeiro (n° 031/2018). Eighteen C57Bl/6 male mice (3 months old) were maintained in a temperature-controlled environment (21 ± 2°C) and controlled by undergoing a reversed light cycle (12-hour light/dark), following the committee's guidelines. The mineral and vitamin contents of both diets were based on AIN-93M; the diets were manufactured by Pragsoluções (Jau, São Paulo, Brazil), and their composition was previously described in Pereira et al. [26]. For eighteen weeks, the animals were subjected to a control diet (C) containing 10% of the total energy from lipids or a high-fat diet (HF) with 60% of the total energy from lipids.

### 2.3. Treatment with Capybara Oil and Experimental Groups

From the 15th to the 18th week (4 weeks), the animals were subdivided. They began to receive daily treatment with a placebo (drinking water) or free capybara oil (CO) (5 g/kg) through orogastric gavage, according to the following experimental groups (*n* = 6/group): control (C), high fat (HF), and high fat treated with capybara oil (HF + CO). The C and HF groups received placebo treatments.

### 2.4. Body Mass and Oral Glucose Tolerance Test

Body mass (BM) and oral glucose tolerance test (OGTT) were measured at the end of the experiment. Mice were weighed on a precision digital scale, and their BM was calculated. The OGTT was performed to assess oral glucose tolerance, for which the animals fasted for eight hours. Blood was collected through a small incision at the tip of the mouse's tail for fasting glucose testing (Bayer, Rio de Janeiro, Brazil); then, a 25% glucose solution (2 g/kg mouse) was administered by gavage, and the plasma glucose concentration was measured at 15, 30, 60, and 120 minutes after the administration of the glucose solution.

### 2.5. Euthanasia

On the day of euthanasia, after 18 weeks of experimentation, the animals were anesthetized with pentobarbital (40 mg/kg) in the intraperitoneal region. Blood samples were extracted by cardiac puncture and centrifuged (1200g, 15 min), and the plasma was removed and stored in a −80°C refrigerator. The liver was collected and fixed in 4% paraformaldehyde for routine histology and immunohistochemistry, or fixed in 2.5% glutaraldehyde for processing for transmission electron microscopy, or frozen in refrigeration at −80°C for analysis of oxidative stress.

### 2.6. Biochemical Assessment

Plasma alanine aminotransferase (AST) and aspartate aminotransferase (ALT) were measured by spectrophotometry performed according to the manufacturer's specifications (Bioclin System II, Belo Horizonte, Brazil). For this analysis, the CMD 800 (Wiener Lab Group, Santa Fe, Argentina) device was used. Calculations for concentrations in g/dL were used according to the manufacturer's protocol.

### 2.7. Morphological Analysis

The liver was collected and fixed with 4% buffered paraformaldehyde. For routine histology, liver samples were washed (3 h) and dehydrated in an increasing series of 70% (24 h), 90% (40 min), and two baths of 100% (40 min each) ethanol, diaphonized in xylol 2 (25 min each), and then infiltrated in paraffin 2 (1 h each). After embedding in paraffin, blocks containing liver samples were sectioned into 3 *μ*m-thick slices. The slices were stained with hematoxylin-eosin (HE) for histopathological analysis of hepatic steatosis; then, the slides were observed under a light microscope equipped with a CCD camera (Olympus BX 53 with Olympus DP72 camera, Tokyo, Japan) and processed in a computerized image capture system, Image Pro-Plus 7.0 (Media Cybernetics, Silver Springs, USA). Five random fields (40x) from each liver slice per animal (*n* = 6) were photographed under the microscope.

### 2.8. NAFLD Scoring

The NAFLD scoring was performed according to Liang et al. [27]. The score was used to calculate progression from NAFLD to NASH. There are two key features of NASH, steatosis and inflammation. Steatosis was determined by analyzing hepatocellular vesicular steatosis, which was divided into macrovesicular steatosis and microvesicular steatosis separately, and by hepatocellular hypertrophy. Inflammation was scored by analyzing the amount of inflammatory cell aggregates, which were considered to only cluster with more than 5 inflammatory cells. The scoring system is shown in Table 1, and the criteria for diagnosis are shown in supplemental material S1. The purpose of this scoring system is to score individual characteristics.

Macrovesicular and microvesicular steatosis were scored separately and graded according to the severity based on the percentage of the total area affected into the following categories: 0 (<5%), 1 (5–33%), 2 (34–66%), and 3 (<66%). Hepatocellular hypertrophy was defined as cellular enlargement more than 1.5 times the normal hepatocyte diameter and was also graded based on the percentage of the total area affected into the following categories: 0 (<5%), 1 (5–33%), 2 (34–66%), and 3 (<66%). The unweighted sum of the scores for steatosis (macrovesicular steatosis, microvesicular steatosis, and hypertrophy) ranged from 0 to 9. Both steatosis and hypertrophy were evaluated at 40x magnification, and only the sheets of hepatocytes were considered. For all the analysis, five different fields per animal were used.

Inflammation was evaluated by counting the number of inflammatory foci per field using 100x magnification. The focus of inflammation was defined as a cluster, not a row, containing more than five inflammatory cells. As well as in steatosis, five different fields per animal were counted, and the average was subsequently scored according to the following categories: normal (<0.5 foci), slight (0.5–1.0 foci), moderate (1.0–2.0 foci), and severe (<2.0 foci).

### 2.9. Immunohistochemistry

For immunohistochemistry, the following primary antibodies were used: anti-Bax (AP00592) (BT LAB, Zhejiang, China), Bcl-2 (AP00611) (BT LAB, Zhejiang, China), and Cytochrome C (Cyto C) (sc 13156) (Santa Cruz Biotechnology, Texas, United States). The slices with the liver sections were deparaffinize and hydrated. The slices were incubated with 3% hydrogen peroxide for 15 min, and the sections were rinsed with distilled water and washed with phosphate-buffered saline with a pH of 7.4 (PBS) three times for 5 min at a time. Antigenic retrieval was performed with a sodium citrate buffer at a pH of 6.0 for 20 min at 60°C. Then, the sections were allowed to cool to room temperature and then washed again with PBS. Subsequently, the sections were incubated with 3% PBS/BSA (bovine serum albumin) for 20 min at room temperature. The sections were then incubated with primary antibodies diluted in PBS at a ratio of 1 : 100 (Bcl-2 and Cyto C) or 1 : 200 (Bax) in a humid chamber overnight at 4°C. The following day, the sections were washed with PBS and incubated with biotinylated secondary antibody for 1 h and then with streptavidin (Dako, Santa Clara, USA) for 30 min at room temperature. After that, the sections were washed again with PBS, revealed with diaminobenzidine (DAB) (Dako, Santa Clara, USA), and counterstained with Harris Hematoxylin. The slices were dehydrated, diaphanized, and mounted with an entellan® mounting medium. Immunoperoxidase expression was performed using the Image-Pro Plus 7.0 program (Media Cybernetics, Rockville, USA) and was measured by quantifying a pixel per area (pixel/*μ*m^2^). Five random fields of each liver fragment per animal were used (*n* = 6) and analyzed with a microscope under 400x magnification for the quantification of immunostaining. The areas marked with DAB, which were more brownish, were manually selected, obtaining the results.

### 2.10. Oxidative Stress Assay

Liver samples were homogenized using a tissue homogenizer in 500 *μ*L of potassium phosphate + EDTA (KPE) buffer (pH 7.5) and then centrifuged at 600 × g for 10 min at 4°C. The supernatant was collected, and the pellet was discarded. In the end, the samples were stored at −20°C until the moment of the analyses, as performed by Pinheiro [22]. Superoxide dismutase (SOD) activity was assayed by monitoring adrenaline (Sigma-Aldrich, St. Louis, Missouri, USA) inhibition and auto-oxidation. The activity is determined by the inhibition of the epinephrine self-peroxidation product during auto-oxidation. For the measurement of SOD activity, glycine buffer (pH 10.2) was used, and the tissue samples were arranged in three different volumes (1 *μ*L, 2 *μ*L, and 3 *μ*L). Then, 193 *μ*L of glycine buffer, 2 *μ*L of catalase, and 4 *μ*L of epinephrine were added, and the reading was performed immediately in a spectrophotometer (SpectraMax M5—Molecular Devices) at 480 nm. As a control, a blank was made, and the free oxidation of epinephrine was evaluated without the samples. Catalase (CAT) activity was measured by a decrease in the H2O2 (Sigma-Aldrich, São Paulo, Brazil) rate, and concentrations were monitored. The activity of the catalase enzyme was determined by the rate of decay of hydrogen peroxide (H2O2) at known concentrations in the first minute of the reaction (1 min). For this assay, “MIX” was prepared, containing 25 mL of distilled water and 40 *μ*L of hydrogen peroxide. Subsequently, 1 *μ*L of the sample was added to 99 *μ*L of the MIX. The samples were read in a spectrophotometer (SpectraMax M5—Molecular Devices) at 240 nm absorbance using a UV plate.

### 2.11. Transmission Electron Microscopic Study

To evaluate the ultrastructural aspects, the liver was cleaved and fixed in a 2.5% glutaraldehyde solution for 48 h. The material was washed in 0.1 M sodium cacodylate buffer and postfixed with 1% osmium tetroxide and 0.8% potassium ferricyanide for 1 h. After washing, the samples were dehydrated in increasing concentrations of acetone (30, 50, 70, 90, and 100%) for 30 min each; then, they were infiltrated with acetone and epon resin (in proportions of 2 : 1 for 2 h, 1 : 1 for 2 h, and 1 : 2 for 2 h and overnight in pure epon resin). On the following day, the samples were inserted in a new pure epon resin, which was polymerized in an oven at 60°C for 3 days. Ultrathin sections (60–70 nm) of selected areas were performed with an ultramicrotome Leica EM UC7 (Wetzlar, Germany) and counterstained with 5% uranyl acetate and lead citrate for observation in the JEM1011 transmission electron microscope (JEOL, Akishima, Tokyo, Japan).

### 2.12. Statistical Analysis

Data are presented as mean ± standard deviation (SD). The program GraphPad Prism version 8.0.1 (GraphPad Software, Boston, USA) was used for the statistical analyses. The data were obtained by comparing groups, and differences between them were tested. A comparison of data in the pretreatment period was performed using the one-way ANOVA test, followed by the Holm Sidak post-test. The significance level used was 3%.

The NAFLD scoring statistics were performed using SPSS version 29 (IBM, Somers, New York, USA). The scoring system was determined by calculating the intraclass correlation coefficient (ICC) to determine interobserver reliability among three observers and using a two-way random model with absolute agreement and the 95% confidence interval (CI). ICCs were calculated for the agreement on the NAFLD/NASH criteria of macrovesicular steatosis, microvesicular steatosis, hypertrophy, and inflammation.

The ICC interpretation was according to the Munro classification system: values below 0.25 were considered with little or no correlation, values between 0.26 and 0.49 with low correlation, values between 0.50 and 0.69 with moderate correlation, values between 0.70 and 0.89 with high correlation, and values between 0.90 and 1.00 with a very good correlation [28]. For better visualization, the correlation between individual measures was analyzed, as was the interobserver reproducibility of the Bland–Altman plot [29], and the results were considered significant at *p* < 0.05.

## 3. Results

### 3.1. Anthropometric Measurement and Biochemical Assessment

For physiological analyses, we evaluated body mass and TOTG at the end of the experiment (week 18), and for liver function, plasma levels of AST and ALT were measured (Table 2).

The BM in the HF and HF + CO groups was significantly higher when compared to the C group. The HF + CO group showed a slight reduction; however, it was not a significant reduction when related to the HF group. As in BM, the HF and HF + CO groups demonstrate a significant increase in blood glucose levels by TOTG analysis when compared to the C group, suggesting that these animals developed possible insulin resistance. However, the HF + CO group showed no difference in relation to the HF group.

AST and ALT are liver function-marker enzymes. In our work, we observed that AST levels were significantly increased in the HF group when compared to the C group. Contrary to the previous parameters, the treatment with CO managed to significantly decrease the AST levels of the HF + CO group compared to the HF group. However, ALT levels did not differ between groups, and the levels detected were the base levels of the equipment.

### 3.2. Histopathological Evaluation

**Figure 1:**
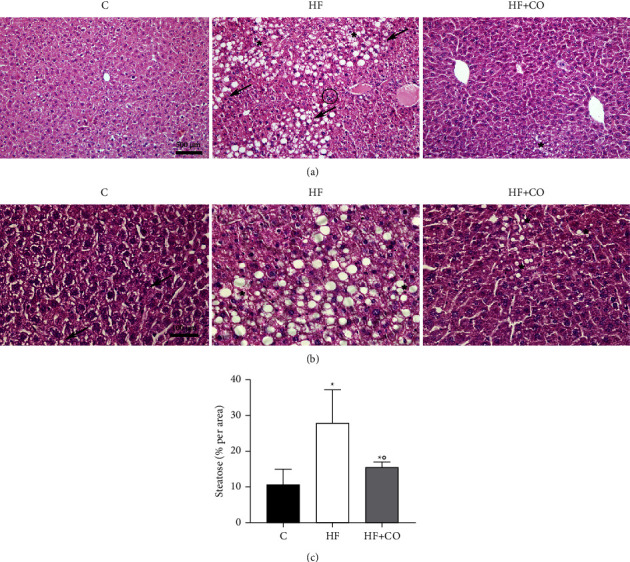
Assessment of the degree of steatosis in the liver. Hematoxylin and eosin staining for the observation of steatosis. (a) HE staining to observe steatosis with a 20x objective. 500 *μ*m calibration bar. (b) HE staining to observe steatosis 40x objective. 50 *μ*m calibration bar. The arrow indicates macrovesicular steatotic areas. The star indicates microvesicular steatotic areas. Circles indicate inflammatory cells. Results are expressed as mean ± standard deviation of the mean. (^*∗*^) *p* < 0.03 compared to group C; (°) *p* < 0.03 compared to the HF group. Statistical analysis: one-way ANOVA with the Holm Sidak post-test. *N* = 6 for all experimental groups.

For histopathological analysis of the liver, HE staining was performed, from which we calculated the NAFLD score and quantified the blank spaces to visualize the percentage of steatosis. With histological analysis, we can observe that in group C, rare lipid droplets are present (arrow) (Figure 1(b)). The HF group shows a large area of hepatocytes exhibiting displaced nucleus, microvesicular (star), and great macrovesicular steatosis (arrow) (Figures 1(a) and 1(b)). It is also possible to visualize some inflammatory cells (circles), which are not present in all animals. Hepatocytes of the HF + CO group presented that microvesicular steatosis has an abnormal accumulation of lipids but with a preserved cellular architecture, including a nondisplaced nucleus (Figures 1(a) and 1(b)). When performing the quantitative analysis (Figure 1(c)), we observed that the percentage significantly increased in the degree of steatosis in the HF group (27.92 ± 9.22) when compared to the C group (10.61 ± 4.34) and that the administration of CO was able to significantly reduce the degree of steatosis in the HF + CO group (15.53 ± 1.37) when compared to the HF group.

**Figure 2:**
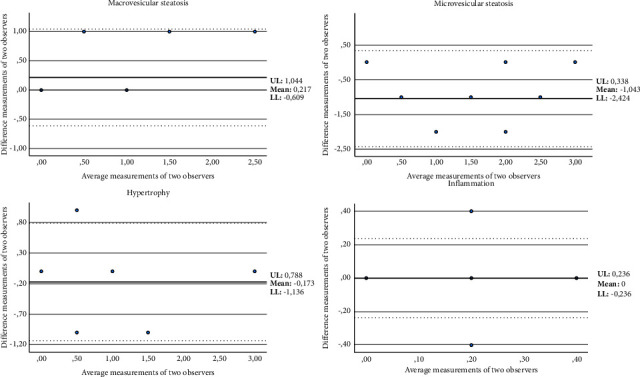
Bland-Altman plot for correlation analysis between two observers for measurement of macrovesicular steatosis, microvesicular steatosis, hypertrophy, and inflammation. *X*-axis: average measurements of two observers. *Y*-axis: difference between measurements of two observers. UL: upper 95% limits of agreement. LL: lower 95% limits of agreement.

To calculate the NAFLD score, a total of 90 images (five from each animal) for each increase in magnification (40x and 100x) were used. To evaluate the agreement between different observers, three different observers analyzed the study set blindly. The level of interobserver variation was subsequently evaluated using the ICC. The ICC indicated a very good correlation for the analysis of macrovesicular steatosis (ICC = 0.909, *p*, 0.001), a moderate correlation for microvesicular steatosis (ICC = 0.690, *p*, 0.001), and a high correlation for the analysis of hypertrophy and inflammation (ICC = 0.797 and 0.888, both *p*, 0.001, respectively) between the different observers (Table 3). In conjunction with the ICC, the Bland–Altman plots for two observers were also reported to visualize the correlation (Figure 2). According to the scoring and visualization of the images obtained, only one animal during the entire experiment progressed to NASH, and this animal belonged to the HF group.

### 3.3. Ultrastructural Analyzes

**Figure 3:**
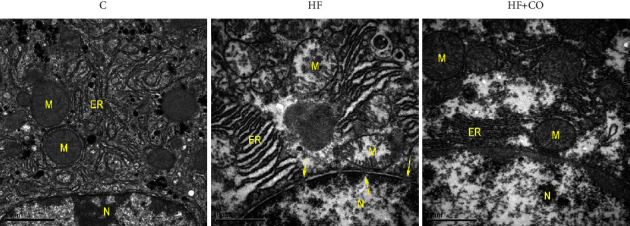
Cross-sectional electron micrographs of the liver of C, HF, and HF + CO groups. M, mitochondria; N, nucleus; ER, endoplasmic reticulum. The arrow indicates pores in the carioteca. Magnification: 20.000x. 1 *μ*m calibration bar. *n* = 3.

In the analysis of the liver ultrastructure by transmission electron microscopy (Figure 3), the animals in the C group showed an ultrastructural organization of hepatocytes with a cytoarchitecture in a well-organized arrangement, showing mitochondria with an intact mitochondrial membrane and preserved endoplasmic reticulum (ER). In the HF group, we see a loss of integrity of the mitochondrial membrane, ER with well-dilated cisterns, and greater disorganization in the cytoarchitecture, in addition to small pores in the carioteca of the nucleus of these groups (arrows); characteristics were reverted with treatment, since, in the HF + CO group, we were able to observe a recovery of the integrity of the mitochondrial membrane and the ER, leading to the absence of visible pores in the nucleus of these animals, in addition to an improvement in the cytoarchitecture of these hepatocytes.

### 3.4. Assessment of Apoptosis by Immunohistochemistry

**Figure 4:**
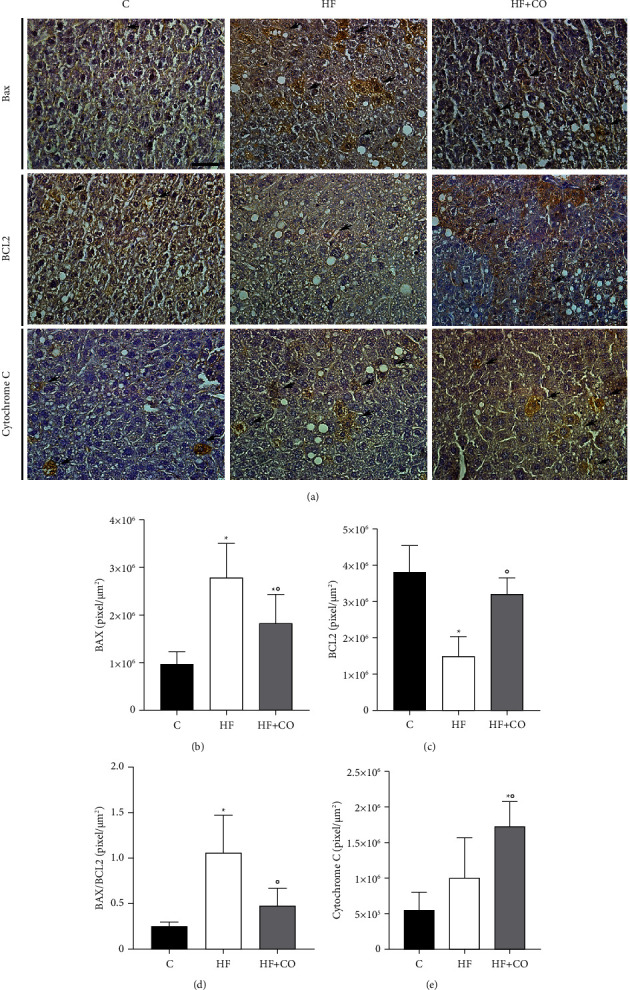
Assessment of the hepatocyte apoptotic pathway. (a) Immunostaining of anti-bax, anti-Bcl2, and anti-cytochrome c antibodies in the liver of experimental groups. 40x objective. 50 *μ*m calibration bar. (b) Graph with quantification of bax levels. (c) Graph with quantification of Bcl-2 levels. (d) Graph with the Bax/Bcl2 ratio. (e) Graph with quantification of cyto c levels. The arrow indicates immunostaining in hepatocytes. Results are expressed as mean ± standard deviation of the mean. (^*∗*^) *p* < 0.03 compared to group C; (°) *p* < 0.03 compared to the HF group. Statistical analysis: one-way ANOVA with the Holm Sidak post-test. *N* = 6 for all experimental groups.

Cell damage was observed through immunostaining of Bax, Bcl-2, and Cytochrome c (Cyto c), which are proteins that participate in the cell apoptotic process. We observed in the C group a significantly lower expression of Bax when compared to the HF and HF + CO groups, which presented greater immunostaining (arrow). The treatment group HF + CO, even though it was still higher than the C group, showed a significantly large decrease relative to the HF group, indicating that the oil increases activation of the proapoptotic pathway in these animals (Figures 4(a) and 4(b)). When evaluating Bcl-2, Group C was significantly more immunostained than the HF group. As before, the HF + CO treatment group was able to reverse the effect caused by obesity, with a significantly higher number of markings when compared to the HF group, conferring an antiapoptotic characteristic in the cells of these groups (Figures 4(a) and 4(c)). We also evaluated the Bax/Bcl-2 ratio (Figure 4(d)). We noticed a significant increase in the HF group when compared to the C group, and the HF + CO treatment group was able to significantly decrease this ratio compared to the HF group. Finally, we evaluated the expression of Cyto c; even though we did not observe a significant result, we could notice an increase in expression in the HF group compared to the C group. In this immunostaining, we saw an unexpected increase in Cyto c expression in the HF + CO group compared to the HF group (Figures 4(a) and 4(e)). Positive and negative controls are provided in supplementary material (S2).

### 3.5. Analysis of the Activity of Enzymes Related to Oxidative Stress

**Figure 5:**
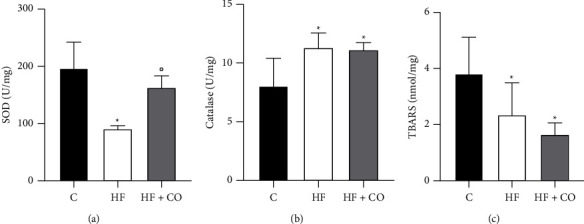
Evaluation of the activity of enzymes related to oxidative stress. (a) Quantification of SOD antioxidant enzyme activity (U/mg). (b) Quantification of the activity of the antioxidant enzyme CAT (U/mg). (c) Quantification of the TBARS technique (nmol/mg). Results are expressed as mean ± standard deviation of the mean. (^*∗*^) *p* < 0.03 compared to group C; (°) *p* < 0.03 compared to the HF group. Statistical analysis: one-way ANOVA with the Holm Sidak post-test. *N* = 6 for all experimental groups.

In the analysis of the enzymatic activity of proteins associated with oxidative stress (Figure 5), we observed that SOD values (Figure 5(a)) were significantly lower in the HF group when compared to the C group; the HF + CO treatment group was able to increase these levels when compared to the HF group. Regarding the CAT (Figure 5(b)), we observed that both the HF group and the HF + CO group were significantly higher than the C group. Finally, we also analyzed the malondialdehyde levels by TBARS (Figure 5(c)), in which we observed the opposite of the CAT, where the HF and HF + CO groups were significantly reduced in relation to the C group.

## 4. Discussion

The administration of a high-fat diet to C57Bl/6 mice for the induction of obesity has already been established in the literature [30, 31]. In the present study, HF mice showed increased BM and oral glucose tolerance levels, indicating possible insulin resistance (IR). Plasma alterations in AST levels could also be observed, in addition to liver changes with increased steatosis and activation of the apoptotic pathway, ultrastructural changes, and an imbalance of antioxidant enzymes.

Many authors have been studying the correlation of obesity with NAFLD and its consequences, such as hepatic steatosis [6, 7]. When the capacity of adipose tissue for energy storage is reduced, the hepatocyte ends up being responsible for functions similar to those of adipocytes, being assigned to these cells' characteristics, such as the storage of lipids, which the adipose tissue was not able to accommodate. In obesity, we can observe an excess of circulating lipids that will be responsible for causing an accumulation of fat, mainly in the liver and skeletal muscle, and IR in multiple organs, with diet as the main way of obtaining these lipids [32–34].

On the other hand, some studies have shown the beneficial correlation of PUFA supplementation for the treatment of obesity and its associated diseases [13, 14]. Work with krill oil (crustacean) and fish oil, both rich in PUFA, has shown positive data from treatment with these oils in animals fed with a high-fat diet [35, 36]. Currently, the most widely used source of PUFA is fish oil [17]; however, seasonal variation, marine pollution, and increased commercialization of omega-3s have created a high demand for this oil, making its use somewhat unsustainable [18]. Therefore, the search for an alternative source of these AGs is essential, and it is in this scenario that capybara oil presents itself. Three studies have already shown the benefits that this capybara-derived by-product can generate, including accelerating the healing process in rat skin wounds; improving steatosis, inflammation, and hepatic mitochondrial activity; and improving pathophysiology and kidney inflammation [24–26].

In the present study, the BM of mice fed with a high-fat diet increased in relation to animals fed with a control diet. Data corroborate the literature, in which we found several works that relate the administration of a high-fat diet with the increase in BM in mice [25, 37]. Furthermore, this type of diet has already been seen to develop consequences such as obesity, hyperinsulinemia, hyperglycemia, hypertension, and liver damage [26, 38].

The large amount of saturated fat in the diet is closely related to the development of IR and elevation of plasma glucose [39], and regardless of being related to obesity, the large amount of saturated FAs has been related to the development of metabolic disorders, such as glucose intolerance and insulin insensitivity [40, 41]. In our data, we saw that the administration of a high-fat diet was responsible for increasing blood glucose levels, suggesting possible IR.

ALT and AST are enzymes located mainly in liver cells and are responsible for catalyzing and transferring amino acids between amino acids and keto acids. When a hepatocyte is injured, its plasmatic membrane will have its permeability increased, and the release of AST and ALT in the blood will occur, making these enzymes important biochemical markers for the evaluation of liver function [42, 43]. There are five main predictors of NAFLD and liver injury. Among them, we find ALT and AST levels, and one study ranked that, among the two enzymes, AST had the best predictive properties for these comorbidities [44]. In our work, although the ALT levels showed no difference, we could observe that the AST levels increased significantly in the HF group compared to the C group.

Treatment with CO could significantly decrease AST levels in HF animals; studies indicate that supplementation with fish oil can decrease both ALT and AST levels in animals with NAFLD [45, 46], and only one study observed the analysis of these enzymes with the capybara oil, in which a positive correlation between treatment and ALT reduction was obtained but did not show significant results concerning AST [22].

In the histological analysis, the HF group showed more intense macro and microvesicular steatosis than the C group. Data corroborate the literature, since several studies have already shown that there is a correlation between obesity and hepatic steatosis [47]. Of all the animals in the studies, only one progressed to NASH, and this animal was in the HF group. In general, hepatic steatosis occurs due to an imbalance in the absorption, production, and export of FAs in the liver. Free FAs can be derived from diet, *de novo* lipogenesis in the liver, and lipolysis from adipose tissue [48], and disruption of one or more of the linked pathways regulating liver function can lead to fat retention intrahepatic, causing steatosis and consequently NAFLD [49, 50]. The liver has FA transporters in its membrane that will be responsible for facilitating the transport of these acids to different cell compartments within the cytoplasm; concomitantly, *de novo* lipogenesis will convert acetyl-CoA into new AGs that can be esterified and stored as TGs; another process that occurs is the oxidation of AGs, which is responsible for reducing intrahepatic levels of fat, a process that occurs mainly in the mitochondria. However, any lipid overload and/or mitochondrial dysfunction will result in the accumulation of a wide variety of lipid species and consequently affect cellular functions [48, 51].

Apoptosis is a natural process that occurs throughout an individual's life. From birth to aging, it is a homeostatic mechanism responsible for maintaining cell populations in tissues. However, it can also occur as a defense mechanism, as in immune reactions or when cells are damaged by disease or harmful agents. It is generally linked with several pathologies, including obesity, and is characterized by nuclear fragmentation, formation of apoptotic bodies, and cell shrinkage [52, 53]. It is mediated by an intracellular signaling cascade that involves a variety of molecules, including caspases, adapter proteins, Bcl-2 family proteins, and apoptosis inhibitor proteins [54]. So far, about 25 genes have been identified in the Bcl-2 family, including the antiapoptotic protein Bcl-2 and the proapoptotic protein Bax. These proteins are essential for being able to determine whether the cell commits to apoptosis or aborts the process [55]. There are two pathways involved in the apoptotic process: (i) the extrinsic pathway—receptor-mediated—and (ii) the intrinsic pathway, also known as the mitochondrial pathway, which leads to the release of Cyto c [56]. In our findings, comparing the HF group and the C group, we observed, in immunostaining for Bax and Bcl-2, a significant increase in Bax levels and the Bax/Bcl-2 ratio in the HF group and a decrease in Bcl-2 in the same group, indicating a possible activation of the apoptotic pathway in these animals.

It is believed that the main mechanism of action of the Bcl-2 family of proteins is the regulation of the release of Cyto c from the mitochondria through the permeability of the mitochondrial membrane [55]. Cyto C is a protein capable of performing a variety of functions, depending on its cellular location and the conditions under which it operates, allowing it to be identified as an “extreme multifunctional” protein [57]. It is a cell life-and-death decision-making molecule, as it can regulate cellular energy supply and apoptosis through tissue-specific post-translational modifications. The intrinsic pathway of the apoptotic process will generate a permeability of the external mitochondrial membrane, consequently releasing several proteins to the cytoplasm, such as Cyto c, thus being able to activate the caspases that have as one of their functions the cleavage of crucial substrates to induce the dismantlement of cells [57, 58]. Several studies indicate that Western diets, usually with high caloric intake, can generate mitochondrial dysfunction and increase the cell's susceptibility to apoptosis [12, 59]. In our study, we did not observe a significant difference in the levels of Cyto c between the C group and the HF group; however, in the transmission electron microscopy, we can observe that the mitochondria of the HF group have their ultrastructure compromised.

Changes in lipid metabolism are related to the NAFLD development process and its progression, affecting organelles such as mitochondria and ER [60]. Marinho et al. [24] have already shown that a high-fat diet is capable of compromising the mitochondrial ultrastructure and disorganizing the cytoarchitecture. Data corroborate our work, in which we could observe not only the loss of integrity of the mitochondrial membrane and a more disorganized cytoarchitecture but also the ER with well-dilated cisterns, indicating a compromised function of these hepatocytes.

In the present study, CO (5 g/kg) was able to reverse the degree of steatosis, improve the ultrastructure, increase Bcl-2 levels, and decrease the Bax and Bax/Bcl-2 ratios in the animals in the HF group. However, we observed a significant increase in Cyto c expression in the HF + CO group.

In the literature, treatment with PUFA is already indicated as effective in improving histopathological changes in rat livers, causing a decrease in the accumulation of hepatic lipids [14, 15], and a study published by our group has already shown the effectiveness of CO in improving steatosis [24] and provided data that corroborate our findings. Another finding by Marinho et al. concerns the ultrastructure of animals on a high-fat diet, in which the study observed that CO recovered mitochondrial integrity and improved cytoarchitecture organization; corroborating our study, the treatment was able to reverse the changes caused in the ultrastructure of animals with a high-fat diet. Regarding Bax levels, Kalogerou et al. [60] showed a decrease in Bax expression in mice with optic atrophy after treatment with omega-3, suggesting a deactivation of the apoptosis pathway. Another study suggested that omega-3 treatment had antiapoptotic effects in the intestinal mucosa of rats, with a decrease in Bcl-2 levels even without changes in Bax levels [61]. Data suggest that the administration of treatments rich in omega-3 has a positive relationship with a decrease in the activation of the apoptotic pathway, decreasing Bax and increasing Bcl-2. Therefore, the use of CO (rich in omega-3) could be a good alternative, as was the case in our study with the administration of treatment in the HF group.

When evaluating the expression of Cyto c, it was expected that it would follow the results of Bax; however, in the HF group, we noticed that the administration of the treatment caused an increase in the expression of this protein. The reason for the increase in Cyto c levels is not known for sure; however, as it is an “extremely multifunctional” protein, it can have several completely different functions [62]. Santucci et al. [56], in their work, say that Cyto c can participate as an effective antioxidant agent inside cells, so we suggest that treatment with PUFA may confer antioxidant properties to this protein.

Mitochondrial dysfunction will impair lipid homeostasis in the liver and lead to the overproduction of reactive oxygen species (ROS) [63]. ROS are a by-product of metabolism and play a large role in the development of obesity, in addition to being associated with NAFLD and playing a role in inducing hepatocyte injury [63, 64]. Oxidative stress is a consequence of an imbalance between ROS and prooxidant and antioxidant enzymes, a factor that causes oxidative cell damage [65]. TBARS consists of a technique that primarily detects levels of MDA, which is a product of lipid oxidation of ROS [66]. Several enzymes can keep ROS levels stable. Among them, we can mention SOD and CAT, and their regulation is important for the treatment of NAFLD [67, 68]. Chen et al. [12] showed that a HF diet can decrease the levels of SOD, GPx, and GSH, which are major antioxidant markers, and data that corroborate our work show SOD downregulation in the HF group. In the present study, we can observe deregulation of oxidative stress enzymes with a decrease in SOD and TBARS and an increase in CAT in the HF group when compared to the C group. The CO treatment was effective only at the SOD level, indicating that it may present an increase of this antioxidant enzyme, which helps to improve the toxicity induced by the high-fat diet.

In conclusion, our findings indicate that CO treatment has effects on liver histopathology, improving hepatic steatosis and decreasing the activation of the apoptotic pathway, in addition to being able to restore the ultrastructure of hepatocytes in obese animals and improve SOD and AST levels. These findings are relevant and suggest that OC has great potential for the development of new therapies for the treatment of obesity and NAFLD.

## Figures and Tables

**Table 1:** Grading system for NAFLD scoring.

Histological features	Score			
	0	1	2	3
Steatosis:				
Macrovesicular steatosis	<5%	5–33%	33–66%	>66%
Microvesicular steatosis	<5%	5–33%	33–66%	>66%
Hypertrophy	<5%	5–33%	33–66%	>66%
Inflammation:				
Number of inflammatory foic/field	<0.5	0.5–1.0	1.0–2.0	>2.0

**Table 2:** Body mass, oral glucose tolerance test, and liver function.

Parameters	Groups		
	C	HF	HF + CO
BM (g)	26.83 ± 1.33	37.71 ± 3.82^*∗*^	34.71 ± 3.59^*∗*^
OGTT (mg/dL)	190.60 ± 55.25	281.67 ± 66.43^*∗*^	251.27 ± 72.85^*∗*^
ALT (UI/L)	5.20 ± 0.45	5.00 ± 0.00	5.30 ± 0.49
AST (UI/L)	158.40 ± 11.59	224.80 ± 35.26^*∗*^	161.80 ± 28.69°

Body mass (BM); oral glucose tolerance test (OGTT); alanine aminotransferase (ALT); aspartate aminotransferase (AST). Results are expressed as mean ± standard deviation of the mean. (^*∗*^) *p* < 0.03 compared to group C; (°) *p* < 0.03 compared to the HF group. Statistical analysis: one-way ANOVA with the Holm Sidak post-test. *N* = 6 for all experimental groups.

**Table 3:** Interobserver reproducibility of NASH histological features according to the NAFLD scoring system.

Histological features	ICC	CI	*P*
Macrovesicular steatosis	0.909	0.800–0.960	<0.001
Microvesicular steatosis	0.690	0.181–0.878	<0.001
Hypertrophy	0.797	0.561–0.910	<0.001
Inflammation	0.888	0.777–0.949	<0.001

ICC: intraclass correlation coefficient (two-way random effects model, with absolute agreement); CI: 95% confidence interval; *P*: level of significance.

## Data Availability

Data will be available upon request.

## Abbreviations

BM: Body mass
OGTT: Oral glucose tolerance test
HE: Hematoxylin-eosin
CAT: Catalase
SOD: Superoxide dismutase
CO: Capybara oil
PUFA: Polyunsaturated fatty acid
ALT: Alanine aminotransferase
AST: Aspartate aminotransferase
M: Mitochondria
N: Nucleus
ER: Endoplasmic reticulum.

## Supplementary Materials

Supplementary Materials S1 Figure: Diagnosis diagram for NAFLD. The two key features of NASH, steatosis (0–9) and inflammation (0–3), were used in the proposed rodent scoring system. S2 Figure: Positive and negative controls for the immunohistochemistry apoptotic pathway. A–C: Histological section of human full-term placenta; 100x objective; and 20-*μ*m calibration bar. D: Histological section of the mouse liver; 40x objective; and 50-*μ*m calibration bar. (A) Immunostaining of anti-Bax. (B) Immunostaining of anti-Bcl2. (C) Immunostaining of anti-Cytochrome c. (D) Negative control.
