# Supplementary material for: Beneficial Effects of Capybara Oil Supplementation on Steatosis and Liver Apoptosis in Obese Mice
Source: J Obes. 2024 May 27;2024:7204607. doi: 10.1155/2024/7204607 (PMC11147678; doi:10.1155/2024/7204607)
Supplement: Supplementary Materials — S1 Figure: Diagnosis diagram for NAFLD. The two key features of NASH, steatosis (0–9) and inflammation (0–3), were used in the proposed rodent scoring system. S2 Figure: Positive and negative controls for the immunohistochemistry apoptotic pathway. A–C: Histological section of human full-term placenta; 100x objective; and 20-μm calibration bar. D: Histological section of the mouse liver; 40x objective; and 50-μm calibration bar. (A) Immunostaining of anti-Bax. (B) Immunostaining of anti-Bcl2. (C) Immunostaining of anti-Cytochrome c. (D) Negative control. [file 7204607.f1.zip › supplemental_material_2.docx]

**Figure S2:**


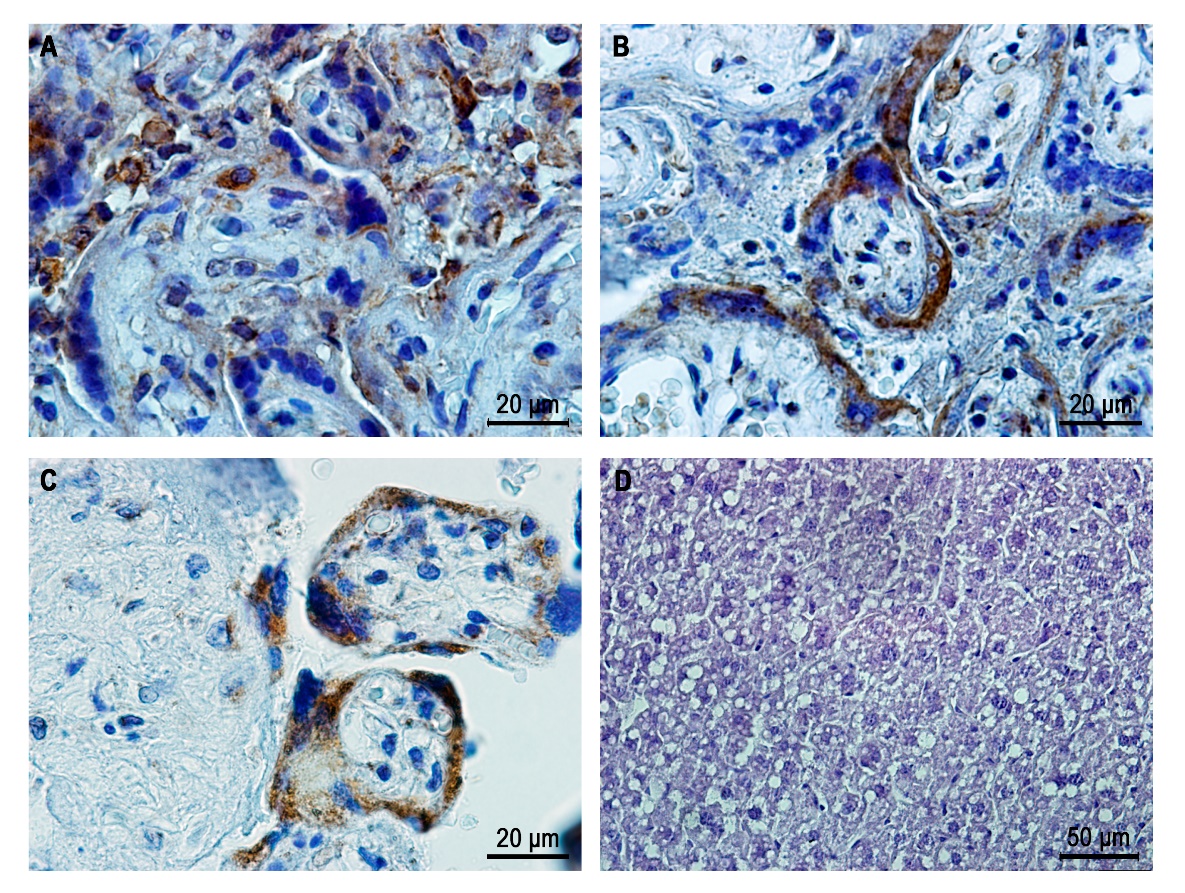


**Legend:** Positives and negative controls for immunohistochemistry apoptotic pathway. A-C: Histological section of human full-term placenta; 100x objective; 20μm calibration bar. D: Histological section of mouse liver; 40x objective; 50μm calibration bar. (A) Immunostaining of anti-Bax. (B) Immunostaining of anti-Bcl2. (C) Immunostaining of anti-Cytochrome c. (D) Negative control.
